# Changes in the Networks of Bedtime Procrastination and Anxiety Symptoms Among Chinese Adolescents

**DOI:** 10.1155/da/7589775

**Published:** 2025-07-15

**Authors:** Tingting Gao, Chengchao Zhou, Yingying Su

**Affiliations:** ^1^Department of Social Medicine and Health Management, School of Public Health, Cheeloo College of Medicine, Shandong University, Jinan, Shandong, China; ^2^NHC Key Lab of Health Economics and Policy Research (Shandong University), Jinan, Shandong, China; ^3^Center for Health Management and Policy Research, Shandong University (Shandong Provincial Key New Think Tank), Jinan, Shandong, China; ^4^School of Public Health, Wannan Medical College, Wuhu, Anhui, China; ^5^School of Public Health and Emergency Management, Southern University of Science and Technology, Shenzhen, Guangdong, China

**Keywords:** adolescents, anxiety, bedtime procrastination, cross-lagged panel design, network analysis

## Abstract

**Objective:** While traditional psychometric approaches, such as latent variable modeling, have primarily focused on the association between bedtime procrastination and anxiety, they often fail to capture symptom-level temporal and directional relationships. Therefore, this study aims to explore the temporal dynamics of symptom-level associations between bedtime procrastination and anxiety, examining both within-person and between-person variations over time in an adolescent population.

**Methods:** This study utilized panel data-based network analyses to examine both within-person effects (temporal and contemporaneous networks) and between-person dynamics across 3,296 adolescents. Specifically, we examined symptom-to-symptom associations of bedtime procrastination and anxiety using both cross-sectional and temporal network analyses and assessed the symptom centrality to identify key drivers of symptom dynamics.

**Results:** At the within-person level, the temporal network analysis indicated that restlessness (GAD5) was the most stable and predictive node across time. Additionally, nervousness (GAD1) and going to bed later than intended (BPS1) had the most significant influence on other symptoms in the T1→T2 and T2→T3 networks, respectively. In the contemporaneous network, inability to control worry (GAD2), excessive worry (GAD3), and trouble relaxing (GAD4) were identified as the central symptoms. At the between-person level, positive relationships between specific bedtime procrastination symptoms were consistently observed.

**Conclusions:** Our findings elucidate the potential complex interactions between bedtime procrastination and anxiety symptoms, highlighting central symptoms that vary across temporal and contemporaneous networks. The identification of central symptoms and their dynamic associations within these networks can inform the causal mechanisms underlying bedtime procrastination and anxiety, thereby guiding the design of targeted interventions for adolescents.

## 1. Introduction

Adolescence, the transitional period between childhood and adulthood, is characterized by significant changes in sleep patterns [1]. Biological factors, such as the maturation of sleep regulatory systems, interact with psychosocial and societal pressures, creating a “Perfect Storm” that leads to insufficient and inappropriately timed sleep [2]. Bedtime procrastination, a novel form of procrastination related to health behaviors, plays a significant role in delayed sleep onset and insufficient sleep [3]. It refers to the intentional delay or failure to go to bed at the intended time, even in the absence of external factors or obstacles [4]. Similar to traditional procrastination linked to unpleasant tasks, individuals who engage in bedtime procrastination may perceive sleep or bedtime routines as equally aversive [4]. In addition to sleep deprivation, bedtime procrastination is associated with a range of negative outcomes, including poor sleep quality, daytime fatigue, insomnia, and mental health problems [5–7].

Adolescents, undergoing substantial physical, cognitive, and social transformations, experience a critical developmental phase marked by heightened vulnerability to anxiety. This vulnerability can manifest in a spectrum of symptoms, ranging from mild and transient to severe forms that significantly impair daily functioning [8, 9]. Anxiety has been consistently found to co-occur with sleep disturbances, a comorbidity that has been well-documented [10]. For example, individuals with insomnia reported significantly higher levels of anxiety than those without sleep difficulties [11], and those with generalized anxiety disorders often exhibit concurrent sleep-related issues [12]. Globally, the prevalence of anxiety-related sleep problems among adolescents is estimated at 7.8%, based on data from 67 countries [13]. While the relationship between general sleep problems and anxiety has been extensively investigated in adolescent populations, there has been less focus on the association between bedtime procrastination and anxiety.

To the best of our knowledge, although the bidirectional relationship between bedtime procrastination and anxiety has been explored, empirical research on this topic among adolescents remains limited [14]. Studies have documented similar associations between bedtime procrastination and anxiety, albeit in different populations, suggesting that this relationship may be relevant across various age groups [15]. In the context of the procrastination health model, bedtime procrastination could contribute to anxiety symptoms through mechanisms such as increased stress and delayed treatment [16]. Procrastinators' emotional distress may be a consequence of their delay patterns, implying that negative moods arise as a result of procrastination rather than serving as its underlying cause [17]. In turn, elevated anxiety led to increased bedtime procrastination [18]. A polysomnographic study revealed that anxiety can impact sleep structure, including sleep onset latency and bedtime procrastination [19]. According to the internalization of conflicts model [20], negative emotions, particularly anxiety, may heighten emotional arousal, resulting in physiological hyperarousal that subsequently contributes to sleep problems, including bedtime procrastination [21, 22]. Bedtime procrastination may function as a maladaptive emotion regulation strategy (e.g., anxiety) in the short term, offering temporary relief from emotional distress, but ultimately reinforcing detrimental sleep habits [23]. Neurobiological evidence further supports the interplay between anxiety and procrastination. The right hippocampus has been identified as a shared neuroanatomical correlate of both trait anxiety and procrastination tendencies [24]. Dysfunction within the hippocampus–prefrontal cortex circuitry may impair the regulation of negative thoughts and emotions in individuals with anxiety, thereby increasing susceptibility to procrastinatory behaviors [25]. However, the majority of existing research has relied on cross-sectional design and latent variable models, which limit the ability to infer causal or temporal directions in the relationships between bedtime procrastination and anxiety. To address this gap, longitudinal network approaches are warranted to uncover the potential bidirectional associations and capture their dynamic symptom-level interactions over time.

Network analysis is a statistical approach used to examine the complex interrelationships among observable variables, particularly individual symptoms of mental disorders [26]. It conceptualizes that symptoms are causally interdependent, whereby each symptom may influence or be influenced by others within a network structure [27]. In this framework, mental disorders emerge from networks of interacting symptoms, with nodes representing symptoms and edges reflecting their statistical associations, often via partial correlations [28]. Several cross-sectional network studies have examined the associations between sleep problems and anxiety symptoms. For example, symptoms such as “nervousness”, “trouble relaxing”, and “uncontrollable worry” have been identified as central or bridging nodes within anxiety-sleep networks [29]. Another study identified “sleep”, “guilt”, “restlessness”, “irritability”, and “feeling afraid” as bridging symptoms linking the network of depression, anxiety, and sleep disturbance [30]. However, undirected cross-sectional networks have important limitations. They cannot account for temporal dynamics or directional influences among symptoms, making it difficult to infer how symptoms evolve over time or which symptoms may trigger others. Additionally, such models often conflate within-person variability, which refers to changes in an individual's symptoms over time, with between-person differences that reflect more stable individual characteristics, thereby limiting their ability to capture dynamic symptom-level processes [31, 32].

To address these limitations, we adopted the cross-lagged panel network (CLPN) models. CLPN combines cross-lagged panel modeling with network analysis to capture temporal and directional relationships between symptoms in longitudinal data [33]. Unlike traditional cross-lagged models focusing on latent variables or total scores, CLPN operates at the symptom level and is better suited to identify central and predictive symptoms across time [34, 35]. Furthermore, CLPN enables the decomposition of within-person and between-person effects, helping to distinguish symptom changes within individuals from average differences between individuals [36, 37]. Within-person effects capture how a phenomenon changes over time within the same individual. In contrast, between-person effects refer to stable differences in how different individuals respond to the same phenomenon [31].

To our knowledge, no prior study has constructed either undirected or directed symptom-level networks to examine the relationships between bedtime procrastination and anxiety in adolescents. In addition, the directionality of these associations at the symptom level has yet to be thoroughly investigated. Most existing network studies have also overlooked the critical distinction between within-person variability and between-subjects differences. To address these gaps, this study aimed to investigate the dynamic relationships between symptoms of bedtime procrastination and anxiety in adolescents over an 18-month period. Specifically, we applied a CLPN model to examine the directionality of symptom-level associations and disentangle within-person dynamics from between-person differences.

## 2. Methods

### 2.1. Study Design and Participants

This study employed a longitudinal design with three waves of data collection over an 18-month period. Data were collected from two junior schools and one senior high school in Shandong Province, China. The first wave of data collection was conducted in November 2021 (T1), followed by the second in May 2022 (T2), and the third in May 2023 (T3). The unequal time intervals between waves (6 months between T1 and T2, and 12 months between T2 and T3) were determined based on both theoretical and logistical considerations. A 6-month interval allowed for capturing short-term symptom fluctuations, while a 12-month interval captured broader developmental changes. This mixed-interval design aimed to balance temporal resolution and explore symptom dynamics across different timescales. A total of 5705 adolescents completed the survey at Time 1 (T1), 3566 at Time 2 (T2), and 3296 at Time 3 (T3). Attrition occurred between the waves primarily due to school transfers, absences on the day of the survey, or incomplete questionnaires. The attrition rate was 37.4% from T1 to T2, and 7.6% from T2 to T3. Comparisons of key demographic characteristics, including age, sex, and family residence, between those who remained in the study and those lost to follow-up indicated no significant differences, suggesting minimal attrition bias.

**Figure 1:**
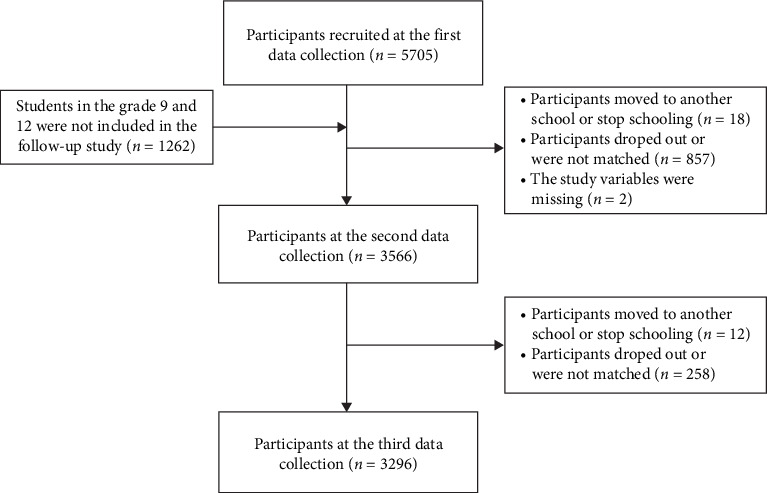
Flow chart of participants included in the analysis.

Finally, 3296 participants who completed both T1 and T3 surveys were included in this study. We selected Time 1 and Time 3 to investigate the long-term developmental patterns of bedtime procrastination and anxiety symptoms over the 18-month span, as these two time points were better suited to capturing more stable and representative changes in symptom trajectories. This approach also helped minimize model complexity and avoid potential overfitting, allowing us to concentrate on the most meaningful temporal relationships. The study sample consisted of 54.5% females (*n* = 1,795) and 65.9% individuals from rural areas (*n* = 2,171). The initial assessment included adolescents aged between 12 and 19 years (*M* = 15.17, SD = 1.44). Participants represented multiple academic levels, with 37.7% in Grade 11 (*n* = 1,243), 35.4% in Grade 10 (*n* = 1,166), 13.5% in Grade 8 (*n* = 445), and 13.4% in Grade 7 (*n* = 442). The sampling procedure used to recruit participants is presented in Figure 1.

Following the approval of the Research Ethics Committee at the School of Public Health, Shandong University (Approval No. LL20210102), the recruitment process for this study commenced. The committee's review ensured that all study procedures adhered to ethical standards for research involving minors. All participants, including minors, as well as their parents or legal guardians, provided written informed consent before participating in this study. This consent indicated their voluntary and informed decision to participate in the study. Importantly, participants were given the autonomy to decide whether to engage in the study and were informed of their right to withdraw at any time without any consequence or obligation. In addition, all paper-based questionnaires were completed in a designated classroom under the supervision of trained research assistants, ensuring a controlled and supportive environment for participants.

Additionally, the privacy and confidentiality of all participant data were strictly maintained throughout the study. To ensure data protection, all personal information was anonymized and stored securely, with access limited to authorized personnel only. Furthermore, participants were informed about the potential risks of the study, and risk mitigation strategies, including providing access to psychological support, were in place to ensure participant safety.

### 2.2. Measures

#### 2.2.1. Bedtime Procrastination

The Bedtime Procrastination Scale (BPS), originally developed by Kroese et al. [3], is a validated and reliable tool for assessing bedtime procrastination behaviors. This 9-item self-report measure utilizes a five-point Likert scale ranging from 1 (never) to 5 (always), with items specifically focused on delaying bedtime beyond intended times (e.g., “I go to bed later than I had intended”). The total BPS score is calculated as the sum of all item scores, with a higher score indicating a greater degree of bedtime procrastination. The BPS has demonstrated satisfactory reliability and validity among various populations, including Chinese university students [38]. In the current sample, Cronbach's *α* coefficients were 0.83 at T1 and 0.85 at T3.

#### 2.2.2. Generalized Anxiety Disorder

The Generalized Anxiety Disorder (GAD-7) is a widely utilized self-reported instrument developed to screen for and assess the severity of anxiety symptoms in both clinical and community populations [39]. The GAD-7 comprises seven items assessing the frequency of anxiety symptoms over the past 2 weeks. Each item is rated on a 4-point Likert scale ranging from 0 (not at all) to 3 (nearly every day), yielding a total score between 0 and 21, with higher scores indicating greater anxiety severity. The GAD-7 has demonstrated strong reliability and validity across diverse cultural contexts, including among adolescent populations [40]. In this study, the measurement exhibited excellent internal consistency, with Cronbach's *α* coefficients of 0.94 at T1 and 0.95 at T3.

### 2.3. Statistical Analyses

Panel data-graphical vector autoregressive model was used to determine three distinct networks: (1) the within-subject temporal network, (2) the within-subject contemporaneous network, and (3) the between-latent network, which captures partial associations for stable trait-level differences over time. This study employed a Gaussian graphical model (GGM) using the graphical Least Absolute Shrinkage and Selection Operator (LASSO) method, combined with an extended Bayesian information criterion (EBIC) for model selection. This regularization technique adjusted the magnitude of regression weights by setting smaller coefficients to zero, thus simplifying the model and enhancing its interpretability. Symptoms were represented as nodes connected by edges, with edge thickness indicating the strength of the connection. Positive associations were shown as blue lines, while negative associations were represented by red lines [41, 42].

To clarify the interrelationships between variables in baseline and follow-up assessments, the CLPN was applied to model time-dependent relationships, controlling for age, sex, and the place of residence [34]. This approach estimates both cross-lagged and autoregressive effects to determine how individual symptoms at one time point predict others at subsequent time points. Autoregressive paths, which emerged as the strongest connections, visually dominated the network and attenuated the visibility of cross-lagged paths. To enhance the interpretability of cross-lagged effects in accordance with the study's aims, all autoregressive paths were constrained to zero [34]. Symptom centrality was assessed using two indices: in-expected influence (IEI) and out-expected influence (OEI). IEI measured how strongly a symptom was influenced by others, while OEI reflected a symptom's ability to predict others at subsequent time points.

Additionally, to disentangle temporal associations at the between-person and within-person levels, a longitudinal network analysis was conducted using a graphical vector autoregression (GVAR) model [43]. To evaluate the accuracy and stability of edge weights and centrality indices, the *bootnet* package was employed, utilizing a nonparametric bootstrapping procedure [44]. The stability of centrality measures was further assessed using the case-dropping bootstrap method, which involves repeatedly estimating the network after excluding up to 70% of the sample. Centrality indices were deemed stable if they exhibited minimal variability across these resamples. Correlation stability (CS) coefficients were also calculated, with values exceeding the recommended threshold of 0.50 interpreted as indicative of strong stability.

The proportion of missing data ranged from 0.5% to 11.2% across variables. To handle missing data, full information maximum likelihood estimation (FIML) was applied, in line with established practices in previous literature [43, 45]. All statistical analyses were conducted using R (version 4.1.1).

## 3. Results

### 3.1. Within-Person Temporal Network

**Figure 2:**
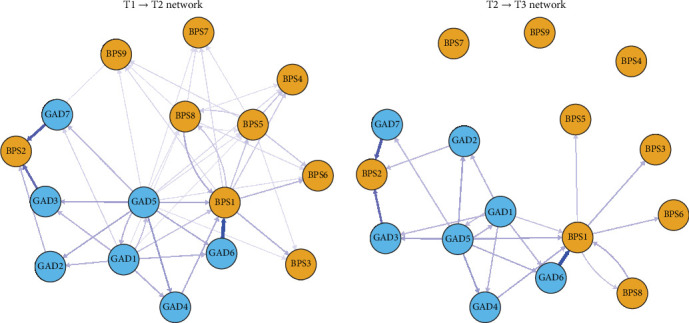
Cross-lagged panel network of bedtime procrastination and anxiety symptoms across three time points: T1→T2 (left) and T2→T3 (right).

**Figure 3:**
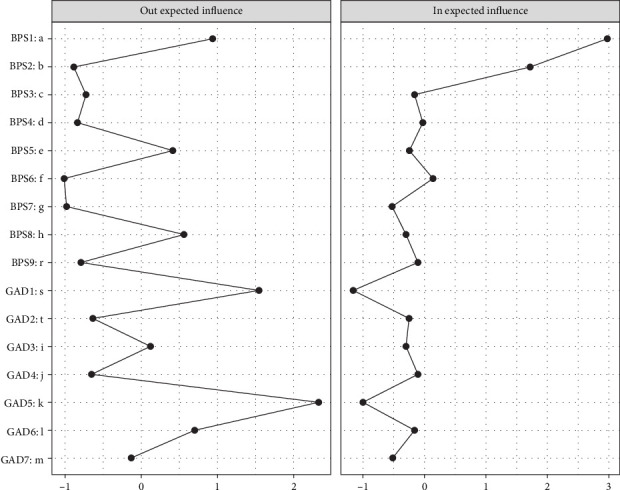
Centrality estimation for the T1→T2 cross-lagged panel network.

**Figure 4:**
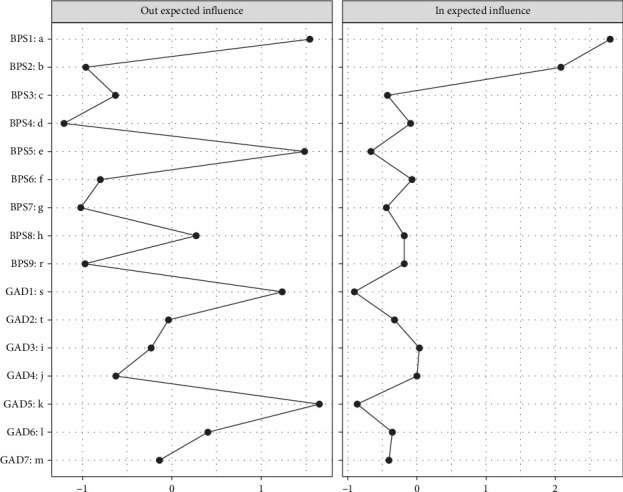
Centrality estimation for the T2→T3 cross-lagged panel network.

Figure 2 illustrates the CLPN, in which edges represent directed effects, and the arrows indicate temporal relationships between symptoms from T1→T2 and T2→T3, respectively. The top three strongest cross-lagged edges included: *irritability* (GAD6) → *go to bed later than intended* (BPS1; *β* = 0.422), *feel afraid* (GAD7) → *unable to sleep early despite early morning wake-up* (BPS2; *β* = 0.303), and *excessive worry* (GAD3) → *unable to sleep early despite early morning wake-up* (BPS2; *β* = 0.242) across the two networks. CLPN centrality estimates were shown across T1→T2 and T2→T3 in Figures 3 and 4. The most impactful nodes with the highest out-prediction and low in-prediction values were *restlessness* (GAD5) and *nervousness* (GAD1) in the T1→T2 network. In addition, the nodes *go to bed later than intended* (*BPS1*) and *unable to sleep early despite an early morning wake-up* (BPS2) had the highest in-prediction values, indicating they were strongly predicted by other symptoms. For the T2→T3 network, the nodes *restlessness* (GAD5) and *go to bed later than intended* (BPS1) had the highest out-prediction values, while the nodes *go to bed later than intended* (BPS1) and *unable to sleep early despite early morning wake-up* (BPS2) had higher in-prediction values. Edge weight difference tests and centrality difference tests are presented in Figures S1–S3. They showed statistically significant differences between most of the identified edge weights and node strengths. The bootstrapping method yielded a relatively narrow edge weight confidence interval (CI) that demonstrated acceptable accuracy (Figure S4). The OEI and IEI centrality demonstrated moderate to strong stability in both T1→T2 (CS-coefficient _OEI_ = 0.750, 95% CI = [0.672,1.000]; CS-coefficient _IEI_ = 0.750, 95% CI = [0.672,1.000]) and T2→T3 networks (CS-coefficient _OEI_ = 0.750, 95% CI = [0.672,1.000]; CS-coefficient _IEI_ = 0.672, 95% CI = [0.595,1.000]).

### 3.2. Within-Person Contemporaneous Network

**Figure 5:**
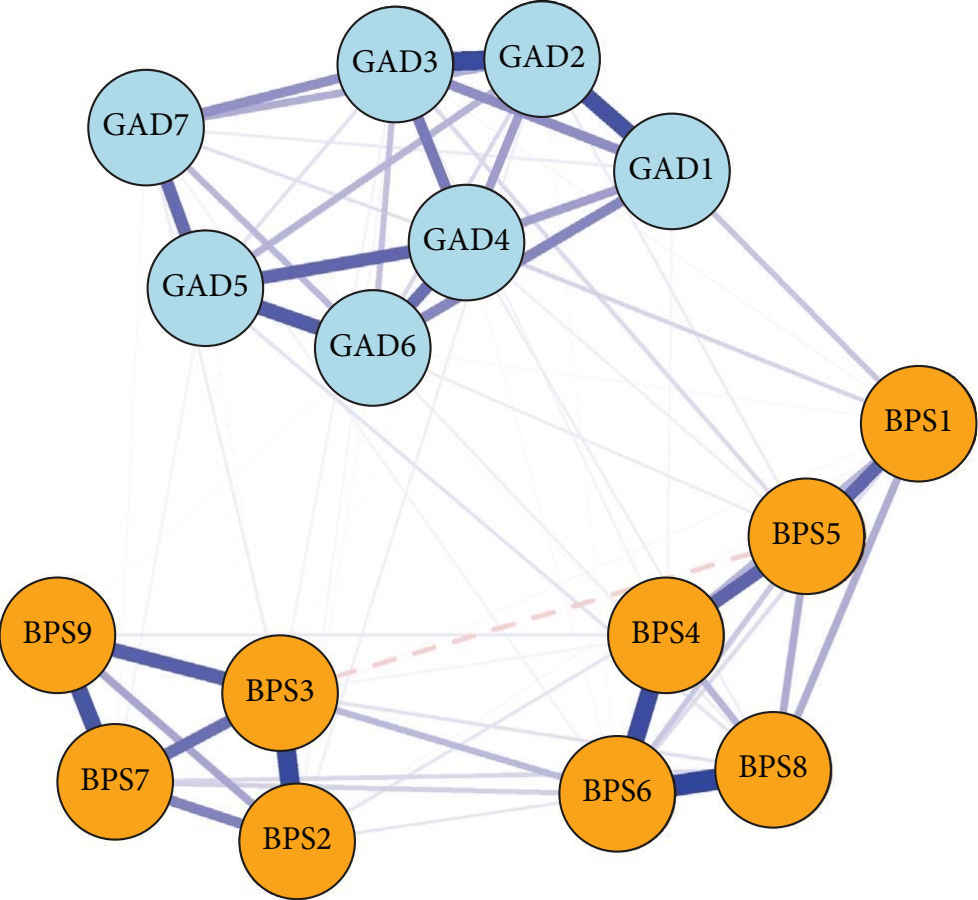
The cross-sectional network of bedtime procrastination and anxiety among adolescents.

**Figure 6:**
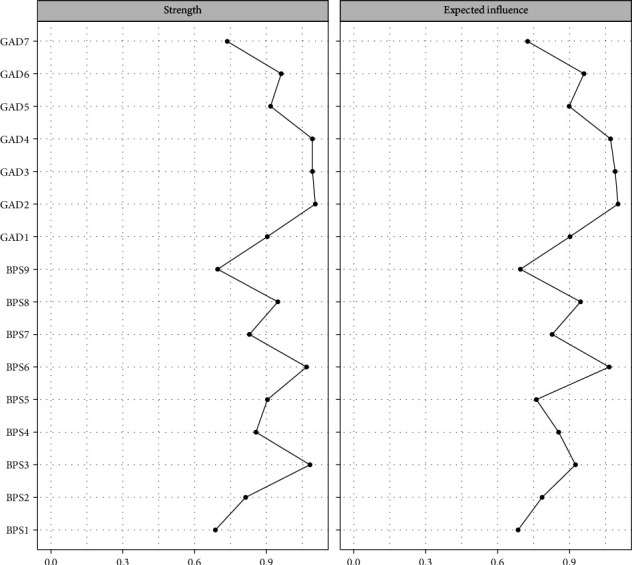
Centrality measures (z-scores) of all symptoms within the network.

The cross-sectional network analysis revealed distinct patterns of associations between symptoms within GAD and BPS domains (Figure 5). In the overall contemporaneous network, the strongest positive association was identified between *delayed bedtime* (BPS6) and *struggle to go to bed on time* (BPS8; *β* = 0.380). Furthermore, significant associations were observed between *inability to control worry* (GAD2) and *excessive worry* (GAD3; *β* = 0.330), as well as between *often procrastinate bedtime with other tasks* (BPS4) and *struggle to go to bed on time* (BPS8; *β* = 0.324).  Figure 6 revealed that the symptom-*inability to control worry* (GAD2) exhibited the highest strength and expected influence centrality, followed by *excessive worry* (GAD3) and *trouble relaxing* (GAD4). The network has sufficient strength correlation stability (CS) coefficients (CS = 0.750 [0.672,1.000]), indicating that the estimated network has high stability and reliability.

### 3.3. Between-Person Network

**Figure 7:**
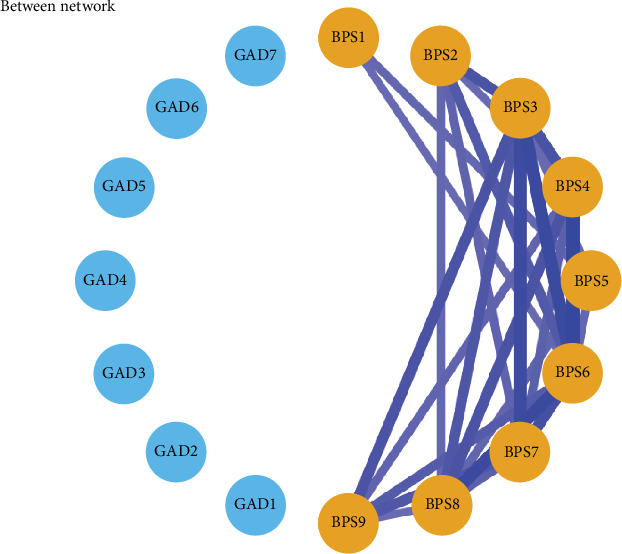
Between-person network of bedtime procrastination and anxiety among adolescents. Edges = partial correlations. Blue edges = positive associations; red edges = negative associations.

The between-person network revealed that *delayed bedtime* (BPS6) exhibited strong positive associations with *reluctance to turn off lights at bedtime* (BPS3), *often procrastinate bedtime with other tasks* (BPS4), and *struggle to go to bed on time* (BPS8) between persons. These findings highlight significant individual differences in the BPS constructs. Details can be found in Figure 7.

## 4. Discussion

This study investigated the dynamic relationships between bedtime procrastination and anxiety symptoms in adolescents using a longitudinal panel network design over 18 months, allowing for exploration of both within-person and between-person symptom interactions across temporal and contemporaneous networks. The use of a CLPN model provides a significant methodological advantage over traditional cross-sectional approaches by capturing temporal precedence, specifically the predictive relationship between fluctuations in one symptom and subsequent changes in another. Our findings identified distinct central symptoms across the temporal and contemporaneous network of bedtime procrastination and anxiety in adolescents. Additionally, the between-person network revealed stable positive associations among specific bedtime procrastination symptoms.

In the within-person temporal network, we found that irritability (GAD6) strongly predicted going to bed later than intended (BPS1). Prior research has identified irritability as a significant predictor of difficulty falling asleep [46], a voluntary sleep-delay behavior resembling the sleep-onset delay typical of delayed sleep–wake phase disorder [47]. Furthermore, both feeling afraid (GAD7) and excessive worry (GAD3) predicted being unable to sleep early despite early morning wake-up (BPS2), suggesting a directional pathway from cognitive-affective anxiety symptoms to disrupted bedtime behaviors. Prior studies also found that “fear” is a bridging symptom within the network of anxiety, depression, and insomnia [48] or in broader sleep disturbance [30]. While insomnia and general sleep disturbances are not synonymous with bedtime procrastination, these findings underscore the potential causal role that cognitive and emotional processes play in contributing to procrastinatory sleep behavior. In addition, increased levels of worry, for example, can lead to muscle tension and negative emotions, disrupting sleep patterns [49]. Physiological hyperarousal near bedtime can also contribute to bedtime procrastination, as adolescents delay sleep to avoid the anticipated difficulty of falling asleep and prolonged wakefulness [18].

While prior literature has hypothesized a possible bidirectional relationship between anxiety and bedtime procrastination, our CLPN findings primarily support a unidirectional temporal association, with anxiety symptoms predicting subsequent increases in bedtime procrastination. This unidirectionality may suggest that anxiety symptoms act as an antecedent to sleep-delay behaviors in adolescents, rather than being directly caused by them. It is possible that effects from bedtime procrastination to anxiety are more subtle, delayed, or dependent on contextual factors that were not captured in our study. Although our temporal network revealed a predominantly unidirectional relationship, we believe this does not preclude a more complex, dynamic interplay between anxiety and bedtime procrastination over time.

Restlessness (GAD5) emerged as the most stable and predictive node across time, while nervousness (GAD1) and going to bed later than intended (BPS1) exerted the strongest influence on other symptoms in the T1→T2 and T2→T3 networks, respectively. Such findings support the network theory of psychopathology, which conceptualizes disorders not as latent categories but as dynamically interacting symptoms. Li et al. [50] also found that “restlessness” (GAD5) is a central symptom within the comorbidity network of depression, anxiety, and insomnia among Chinese college students over time. In a similar pattern, “nervousness” was identified as one of the most central and bridging symptoms within the network of anxiety and sleep disturbances [29]. While prior research focused primarily on insomnia or broader sleep disturbances, our findings emphasize the unique role of bedtime procrastination as a self-regulatory failure with distinct psychological underpinnings, reinforcing its potential as a target for behavioral interventions aiming to disrupt maladaptive affective-sleep feedback loops. Furthermore, consistent with our study, prior research has identified that going to bed later than intended (BPS1) may serve as a key behavioral driver that perpetuates insufficient sleep and contributes to the progression of anxiety symptoms [51].

In the overall within-person contemporaneous network, delayed bedtime (BPS6) was strongly and positively associated with struggling to go to bed on time (BPS8). These symptoms likely reflect challenges in managing self-regulation [52], as individuals with compromised self-regulatory abilities are more prone to engage in bedtime procrastination, often resulting in failure to adhere to planned sleep schedules [4]. Our study findings also align with prior network analyses that identified a strong connection between inability to control worry (GAD2) and excessive worry (GAD3) [53]. These symptoms are central to the anxiety network, where uncontrollable worry tends to exacerbate excessive thinking, creating a feedback loop that can further intensify anxiety. Furthermore, we observed that often procrastinating bedtime with other tasks (BPS4) was closely related to struggling to go to bed on time (BPS8). This pattern echoes qualitative research, which reported that adolescents often face difficulties in managing multiple post-school activities, leading to late-night delays in completing tasks. Consequently, they tend to prioritize other activities, such as social networking, media consumption, and schoolwork, over sleep [54]. In addition, inability to control worry (GAD2), excessive worry (GAD3), and trouble relaxing (GAD4) emerged as central symptoms within the network. Similarly, in the anxiety-sleep disturbance network model, “nervousness,” “uncontrollable worry,” and “trouble relaxing” were identified as central and bridging symptoms in outpatients from high-altitude regions [29]. These symptoms play a crucial role in both the treatment of comorbid anxiety and sleep problems, such as bedtime procrastination, as well as in the development of interventions targeting these interconnected issues. Additionally, the interaction between anxiety and bedtime procrastination may shift depending on whether the symptoms are more trait-like or state-like. For instance, trait-like symptoms, such as chronic worry or habitual procrastination tendencies, may serve as a stable foundation for the development of more acute state-like fluctuations, for example, short-term spikes in anxiety or procrastination during stressful periods. This highlights the need for interventions that address both the trait-like characteristics that may predispose individuals to these behaviors and the state-like variations that could be targeted in times of high emotional distress.

The positive relationships between certain items remained notably consistent at the between-person level, revealing significant individual differences within the constructs of the BPS. Specifically, a strong positive association was found between delayed bedtime (BPS6) and reluctance to turn off lights at bedtime (BPS3). This suggests that individuals who struggle with bedtime procrastination are also likely to experience difficulties in managing environmental cues associated with sleep onset. Additionally, there was a notable connection between often procrastinating bedtime with other tasks (BPS4), and struggling to go to bed on time (BPS8) at the between-person level, which corresponds with patterns observed in the within-person contemporaneous network. These findings are consistent with previous research that has highlighted the role of self-regulation difficulties and external distractions in bedtime procrastination [4]. Specifically, studies have shown that individuals who have trouble managing bedtime often engage in other activities, such as using electronic media or completing tasks, which further delay their sleep onset [5].

Our study has significant value for both theoretical frameworks and practical applications. Different from prior studies that predominantly focused on cross-sectional research and between-person psychometric evaluations, network approaches allow for the simultaneous investigation of multiple variables at both the within-person and between-person levels. By applying a longitudinal panel network approach, this study offers novel theoretical perspectives into how the relationships between bedtime procrastination and anxiety symptoms evolve over time, both across individuals and within individuals. This approach advances contemporary psychopathology theory by shifting the analytical focus from latent variable models, which assume symptoms are interchangeable indicators of underlying constructs, to dynamic systems models, where symptoms are seen as interacting elements capable of causally influencing each other. Our findings highlight the theoretical relevance of identifying central symptoms as potential drivers of symptom escalation or comorbidity, thereby contributing to the symptom network theory framework. In particular, identifying central nodes that connect behavioral and emotional domains may help explain the developmental emergence of internalizing comorbidities in adolescents.

The current findings also carry important clinical and intervention implications. The accumulation of multiple risk symptoms related to bedtime procrastination and anxiety may reinforce self-sustaining feedback loops, where the activation of one or more symptoms could amplify the overall risk. Given the strong interconnections among specific symptoms within the network, addressing one risk symptom, particularly central nodes, may confer protective effects on related symptoms. For instance, interventions targeting cognitive symptoms such as excessive worry and uncontrollable thoughts may alleviate both anxiety and maladaptive bedtime behaviors. Behavioral interventions aimed at enhancing self-regulation and sleep hygiene, including the establishment of consistent bedtime routines, reduction of screen time before sleep, and application of cognitive–behavioral strategies, may effectively disrupt these maladaptive cycles. Moreover, early identification and targeting of central symptoms like restlessness and nervousness may serve as a preventive strategy for adolescents at elevated risk of anxiety and bedtime procrastination. In terms of intervention delivery, school-based programs should adopt an integrative approach that combines behavioral regulation techniques with emotional regulation strategies to address both behavioral and emotional vulnerabilities. These combined approaches are likely to be more effective than single-target interventions. Additionally, the stable associations observed in the between-person network, such as persistent links between task-related procrastination and difficulties initiating sleep routines, emphasize the need for long-term behavioral modifications that target habitual patterns and environmental triggers. Such trait-like associations suggest that individualized, habit-based interventions may yield sustained improvements in both sleep and emotional well-being.

While this study provides valuable insights into adolescent bedtime procrastination and anxiety prevention, it is important to acknowledge its limitations. Firstly, employing unequal time intervals (6 months between T1–T2 vs., 12 months between T2–T3) to assess predictive and causal relationships may influence the interpretation of temporal associations and symptom dynamics. Variations in time lags can affect the strength and direction of observed relationships, making it more challenging to determine the optimal lag for capturing true causal effects. Future research should consider exploring more consistent or alternative interval structures to clarify how timing influences network relationships and symptom evolution. Secondly, the reliance on self-reported measures, which are susceptible to recall bias and social desirability, highlights the need to incorporate multiple data sources, such as observations, informant reports, and physiological markers. Thirdly, due to the regional focus of the study, there is a need for cross-cultural and cross-population validations to assess the generalizability of the findings. For example, although we attempted to control for external contextual variability by collecting data during routine academic weeks and avoiding examination periods, we cannot entirely exclude the potential influence of unmeasured external events. Additionally, pandemic-related stress or socio-environmental changes may have differentially impacted participants across waves, potentially confounding the observed longitudinal patterns in sleep and anxiety. Future research should validate the findings through cross-cultural and cross-population studies, incorporating more comprehensive contextual variables to further explore the impact of external factors on psychological symptom networks. Lastly, the Grade 9 and Grade 12 students were excluded due to the unique academic pressures these students face, such as preparation for the High School Entrance Examination and National University Entrance Examination. These pressures could influence sleep patterns and anxiety, potentially confounding the results. While this exclusion helped focus on a more typical adolescent sample, it may limit the generalizability of the findings. Future studies should consider including these groups to elucidate potential variations in the relationship between bedtime procrastination and anxiety.

## 5. Conclusions

To the best of our knowledge, this study is the first to apply a longitudinal network analysis approach to explore the dynamic, symptom-level interactions between bedtime procrastination and anxiety symptoms in adolescents. By distinguishing within-person fluctuations from between-person differences, this approach enables more precise insights into how specific symptoms temporally influence each other over time at both individual and group levels. It extends the psychometric network approach in psychopathology to enhance our understanding of their dynamic mechanisms. We identified the association between irritability (GAD6) and going to bed later than intended (BPS1), between feeling afraid (GAD7) and being unable to sleep early despite early morning wake-up (BPS2), as well as between excessive worry (GAD3) and being unable to sleep early despite early morning wake-up (BPS2). Restlessness (GAD5) was the most stable and predictive node across time. Targeting the most influential and temporally predictive symptoms may enhance the effectiveness of intervention strategies. By utilizing longitudinal psychometric network models, this study provides a methodological advancement in delineating the dynamic, symptom-level interplay between bedtime procrastination and anxiety. Importantly, this approach facilitates the disentanglement of within-person temporal processes from between-person differences, thereby providing a refined framework for identifying key targets for prevention and early intervention in adolescent populations.

## Data Availability

The data that support the findings of this study are available from the corresponding author upon reasonable request.

## Supporting Information

Supporting Information Figure S1. Edge weight difference tests for the networks using T1 to predict T2 and using T2 to predict T3, respectively. Black boxes indicate edges that significantly differ (*p* < 0.05), and gray boxes indicate edges that do not significantly differ. Figure S2. Estimation of edge weight difference by bootstrapped difference test (T1→T2). Black boxes represent a significant difference. Gray boxes represent the edges that do not significantly differ from one another. Figure S3. Estimation of edge weight difference by bootstrapped difference test (T2→T3). Black boxes represent a significant difference. Gray boxes represent the edges that do not significantly differ from one another. Figure S4. Stability of the centrality indices in the CLPN for T1→T2 and T2→T3.
