# Supplementary material for: Changes in the Networks of Bedtime Procrastination and Anxiety Symptoms Among Chinese Adolescents
Source: Depress Anxiety. 2025 Jul 15;2025:7589775. doi: 10.1155/da/7589775 (PMC12283202; doi:10.1155/da/7589775)
Supplement: Supporting Information — Figure S1. Edge weight difference tests for the networks using T1 to predict T2 and using T2 to predict T3, respectively. Black boxes indicate edges that significantly differ (p < 0.05), and gray boxes indicate edges that do not significantly differ. Figure S2. Estimation of edge weight difference by bootstrapped difference test (T1→T2). Black boxes represent a significant difference. Gray boxes represent the edges that do not significantly differ from one another. Figure S3. Estimation of edge weight difference by bootstrapped difference test (T2→T3). Black boxes represent a significant difference. Gray boxes represent the edges that do not significantly differ from one another. Figure S4. Stability of the centrality indices in the CLPN for T1→T2 and T2→T3. [file 7589775.f1.docx]

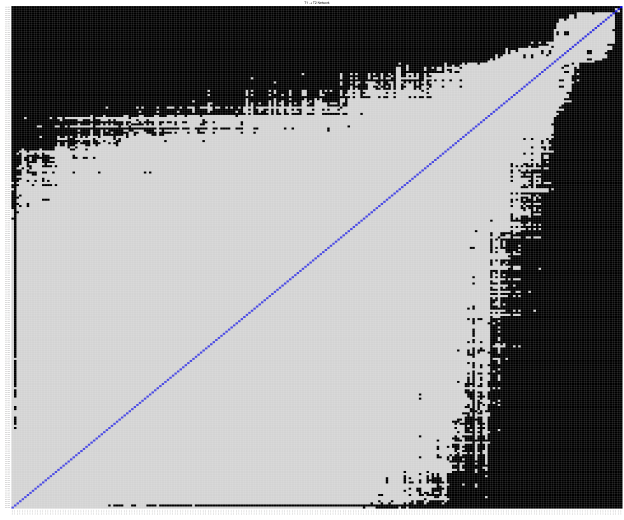

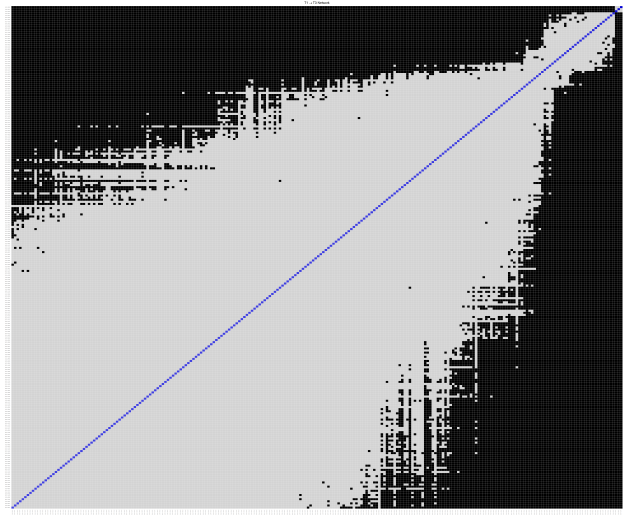


(A) T1→T2 estimation (B) T2→T3 estimation

**Figure S1.** Edge weight difference tests for the networks using T1 to predict T2 and using T2 to predict T3, respectively. Black boxes indicate edges that significantly differ (p < 0.05), and gray boxes indicate edges that do not significantly differ.


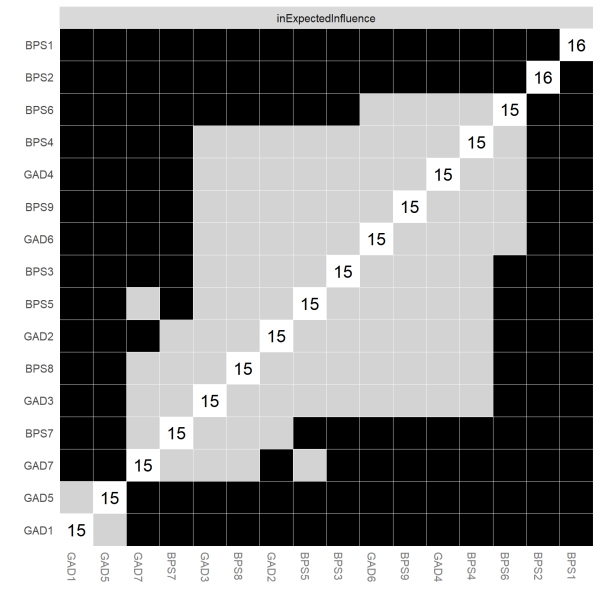

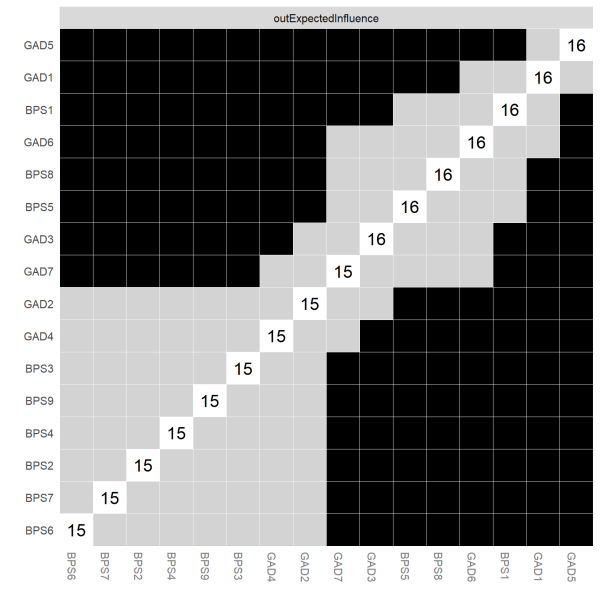


**Figure S2.** Estimation of edge weight difference by bootstrapped difference test (T1→T2). Black boxes represent a significant difference. Gray boxes represent the edges that do not significantly differ from one another.


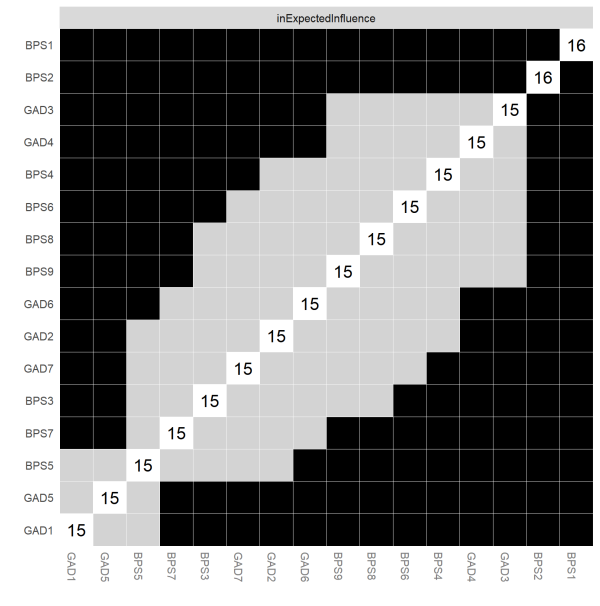

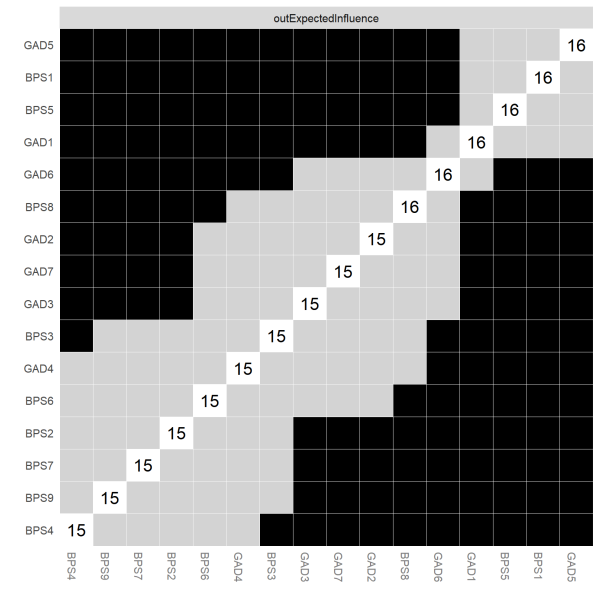


**Figure S3.** Estimation of edge weight difference by bootstrapped difference test (T2→T3). Black boxes represent a significant difference. Gray boxes represent the edges that do not significantly differ from one another.


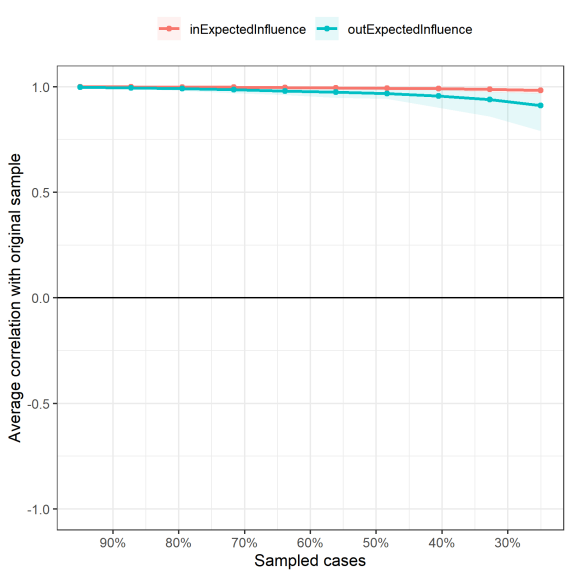

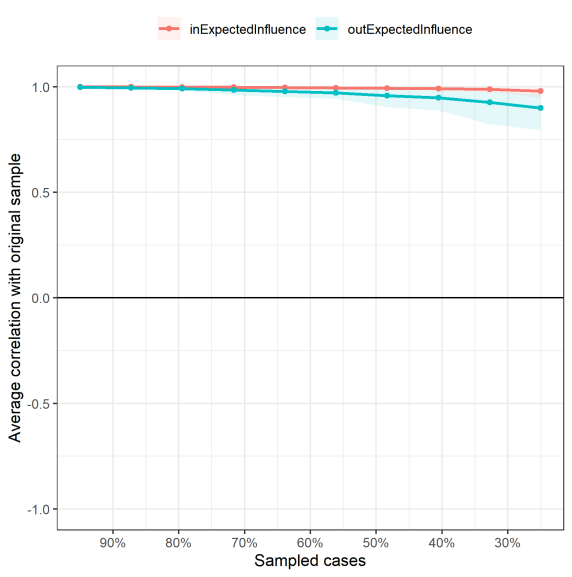


(A) T1→T2 (B) T2→T3

**Figure S4.** Stability of the centrality indices in the CLPN for T1→T2 and T2→T3.
